# 

**Published:** 1895-07-06

**Authors:** 


ABSOLUTELY PURE, therefore BEST.

**CADBURY'S COCOA**

"Of absolute purity and freedom from alkali." —*Braithwaite.*

**CADBURY'S COCOA**

"The Typical Cocoa of English Manufacture - Absolutely Pure."—*The Analyst.*

### THE HOSPITAL

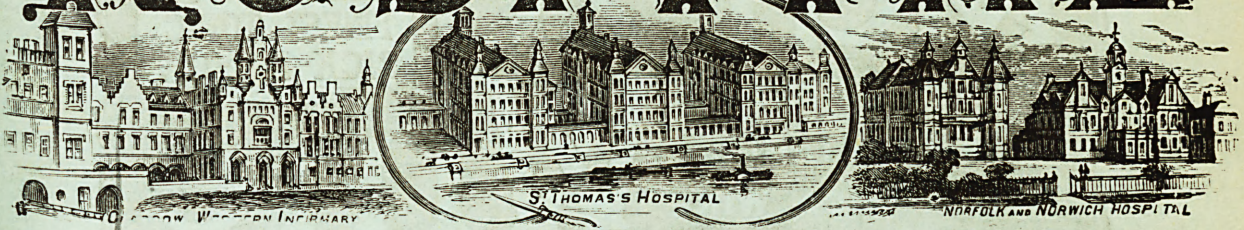

No. 458. Vol. XVIII.

SATURDAY, JULY 6TH, 1895.

WITH EXTRA NURSING SUPPLEMENT.
[Registered at the General Post Office as a Newspaper for transmission in the United Kingdom and Abroad.]

PRICE TWOPENCE.

Per Annum, 10s. 6d., post free.

Monthly Parts, 6d.; post free, 8d.

Ditto per Annum, 8s. 6d., post free.

